# Ablation of KDM2A Inhibits Preadipocyte Proliferation and Promotes Adipogenic Differentiation

**DOI:** 10.3390/ijms22189759

**Published:** 2021-09-09

**Authors:** Yonglin Hua, Yongqi Yue, Dan Zhao, Yan Ma, Yan Xiong, Xianrong Xiong, Jian Li

**Affiliations:** 1 Key Laboratory of Qinghai-Tibetan Plateau Animal Genetic Resource Reservation and Utilization, Ministry of Education, Southwest Minzu University, Chengdu 610041, China; huayonlin11164041@163.com (Y.H.); yyq08053611@163.com (Y.Y.); zd08042@126.com (D.Z.); yanma_199702@163.com (Y.M.); xianrongxiong@163.com (X.X.); 2 College of Animal & Veterinary Sciences, Southwest Minzu University, Chengdu 610041, China; 3 Key Laboratory of Animal Science of National Ethnic Affairs Commission of China, Southwest Minzu University, Chengdu 610041, China

**Keywords:** KDM2A, fat deposition, preadipocytes, proliferation, differentiation, TNPO1, PPARγ

## Abstract

Epigenetic signals and chromatin-modifying proteins play critical roles in adipogenesis, which determines the risk of obesity and which has recently attracted increasing interest. Histone demethylase 2A (KDM2A) is an important component of histone demethylase; however, its direct effect on fat deposition remains unclear. Here, a KDM2A loss of function was performed using two unbiased methods, small interfering RNA (siRNA) and Cre-Loxp recombinase systems, to reveal its function in adipogenesis. The results show that the knockdown of KDM2A by siRNAs inhibited the proliferation capacity of 3T3-L1 preadipocytes. Furthermore, the promotion of preadipocyte differentiation was observed in siRNA-treated cells, manifested by the increasing content of lipid droplets and the expression level of adipogenic-related genes. Consistently, the genetic deletion of KDM2A by Adipoq-Cre in primary adipocytes exhibited similar phenotypes to those of 3T3-L1 preadipocytes. Interestingly, the knockdown of KDM2A upregulates the expression level of Transportin 1(TNPO1), which in turn may induce the nuclear translocation of PPARγ and the accumulation of lipid droplets. In conclusion, the ablation of KDM2A inhibits preadipocyte proliferation and promotes its adipogenic differentiation. This work provides direct evidence of the exact role of KDM2A in fat deposition and provides theoretical support for obesity therapy that targets KDM2A.

## 1. Introduction

Obesity has gradually developed into a global epidemic, and, according to the World Health Organization (WHO), its prevalence has tripled over the last 40 years [1]. This has resulted in the risk and incidence probability of obesity-related metabolic diseases increasing sharply, including type 2 diabetes (T2D), metabolic syndrome, cardiovascular disease and cancer [2]. Thus, the rising cost of living due to the prevalence of obesity and the cost of its prevention makes it necessary to find and develop more effective strategies to prevent and treat obesity [3].

It is well known that obesity is mainly caused by a long-term imbalance between a high energy intake and low energy expenditure, which leads to excessive energy being stored as lipid droplets in adipose tissue [3]. Adipose tissue, as a multifunctional organ, participates in many important physiological activities in vivo [4]. Based on the significant differences in structural characteristics, ultrastructural characteristics, gene expression profiles and biological functions, there are three different types of adipose tissues in animals, namely white adipose tissue (WAT), brown adipose tissue (BAT) and beige/bright adipose tissue (BeAT) [5]. Of these, BAT and BeAT primarily consume fat and convert energy to heat through the mitochondrial uncoupling protein 1 (UCP1), which plays an important role in adaptive heat production [6]. Conversely, WAT mainly stores energy in the form of triglycerides (TG), which leads to white adipocyte excessive expansion and subsequently induces inflammation through paracrine and endocrine avenues [7]. In recent years, a growing number of studies have found that adipogenesis is a complex interplay of multiple molecular determinants and pathways, including transcriptional factors (PPARγ, C/EBPα, C/EBPβ), secretory factors (leptin, adiponectin) and epigenetic regulators, etc. [8,9]. Compared with other factors, the epigenetic regulators that are involved in this process are often far from clear.

Thus, epigenetic regulation has attracted increasing interest as a novel player; for example, histone modifications (methylation, ubiquitination, glycosylation, etc.), which usually affect gene expression by regulating the structure of chromatin [10,11]. Histone methylation is dynamically regulated by the lysine methyltransferase (KMTS) and lysine demethylase (KDMS) families [12]. Among them, histone demethylase 2A (KDM2A) is the first discovered demethylase containing a JmjC domain [13]. Recently, an increasing number of studies have shown that KDM2A is highly expressed in most tumors, except for prostate cancer [14,15,16]. However, its specific role in adipocyte proliferation and differentiation remains unknown.

Here, we measured the expression patterns of KDM2A in WAT from 45 d, 2- and 5-month-old mice, as well as during primary adipocyte differentiation. Additionally, we investigated the role of KDM2A regulation on 3T3-L1 preadipocyte proliferation and differentiation using both the knockdown and knockout techniques. The results indicate that the KDM2A loss of function inhibited preadipocyte proliferation, as assessed by CCK-8 assay, Ki67 and Edu staining, and promoted the preadipocyte differentiation indicated by an increase in lipid accumulation and an elevation of the expression of adipogenic-related genes. Our findings provide new knowledge on the role of KDM2A in adipose tissue and further expand the understanding of the epigenetic gene network that regulates fat deposition.

## 2. Results

### 2.1. Expression Pattern of Histone Demethylase 2A (KDM2A) in White Adipose Tissues at Different Stages

**Figure 1:**
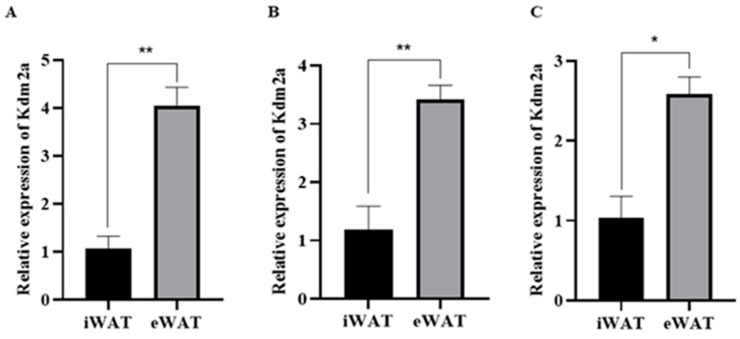
Expression pattern of KDM2A in white adipose tissues at different stages. (**A**) The mRNA level of KDM2A in inguinal white adipose tissue (iWAT) and epididymal white adipose tissue (eWAT) from 45 d mice. (**B**) The mRNA level of KDM2A in iWAT and eWAT from 2-month-old mice. (**C**) The mRNA level of KDM2A in iWAT and eWAT from 5-month-old mice. N = 4; data are presented as mean ± SEM, * *p* < 0.05, ** *p* < 0.01.

Previous studies have found that histone demethylase 2A (KDM2A) is widely expressed in various tissues of mice, including adipose tissue [17]. However, its role in adipose tissue and adipocyte differentiation remains unknown. To reveal the potential effects of KDM2A, we first analyzed the relative mRNA level of KDM2A in white adipose tissue (WAT) from 45 d, 2- and 5-month-old mice using qRT-PCR, including inguinal white adipose tissue (iWAT) and epididymal white adipose tissue (eWAT), which are the classical subcutaneous WAT (SAT) and visceral WAT (VAT), respectively. The results show that KDM2A was expressed in both iWAT and eWAT. In addition, the mRNA level of KDM2A in eWAT was significantly higher than that in iWAT at different stages (Figure 1).

### 2.2. Expression Pattern of KDM2A during Adipogenic Differentiation

**Figure 2:**
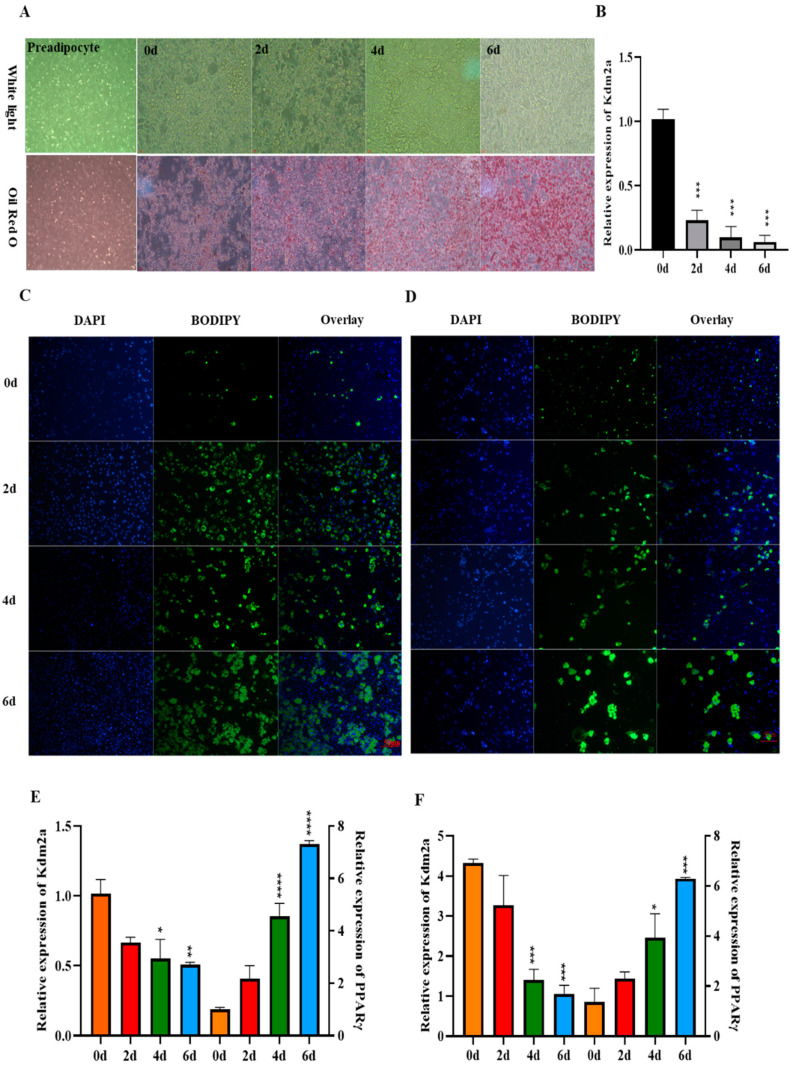
The mRNA level of KDM2A during adipogenic differentiation of 3T3-L1 preadipocytes and primary adipocytes. (**A**) The images of 3T3-L1 preadipocytes and adipogenic differentiation at days 0, 2, 4 and 6. (**B**) The mRNA level of KDM2A during 3T3-L1 preadipocytes adipogenic differentiation. (**C**,**D**) The BODIPY staining of inguinal white adipocytes (**C**) and epididymal white adipocytes (**D**) at day 0, 2, 4 and 6 after adipogenic differentiation. Blue fluorescence labeling of nucleus and green fluorescence labeling of lipid droplets, scale bar: 50 µm. (**E**,**F**) The mRNA levels of KDM2A and PPARγ during inguinal white adipocytes (**E**) and epididymal white adipocytes (F) adipogenic differentiation. N = 4, the number of samples are biological replicates. The data are presented as mean ± SEM, * *p* < 0.05, ** *p* < 0.01, *** *p* < 0.001, **** *p* < 0.0001.

Next, we analyzed the mRNA level of KDM2A during the differentiation of 3T3-L1 adipocytes and primary adipocytes from both iWAT and eWAT. Oil Red O staining was performed to monitor the adipogenesis morphology at day 0, 2, 4 and 6 post-adipogenic induction, and the images show that the accumulation of lipid droplets gradually increased with adipocyte differentiation (Figure 2A). The qPCR analysis showed that the mRNA level of KDM2A exhibited a significant decreasing trend during 3T3-L1 adipocyte differentiation (Figure 2B). Additionally, BODIPY staining was carried out to track the lipid droplet formation in primary inguinal (Figure 2C) and epididymal differentiated adipocytes (Figure 2D) at day 0, 2, 4 and 6. The images show that the BODIPY-positive cells were gradually elevated during adipogenesis and indicate that the differentiation model of primary adipocytes was correct (Figure 2C,D). Consistently, the mRNA level of the differentiation marker gene PPARγ was significantly upregulated in this process (Figure 2E,F, *p* < 0.01), while the expression of KDM2A showed a downward trend (Figure 2E,F). These results demonstrate the level of KDM2A to show a gradual trend of decrease during adipogenesis.

### 2.3. The Subcellular Location of KDM2A In Vivo and In Vitro

**Figure 3:**
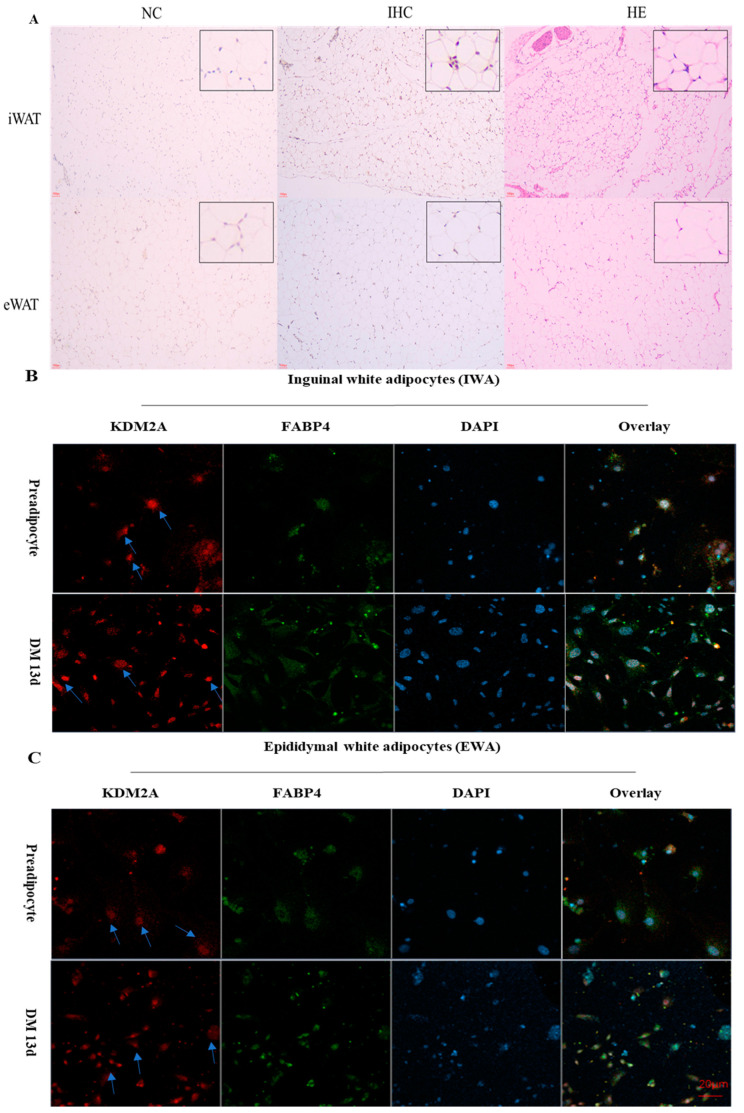
KDM2A is localized in the nucleus. (**A**) Immunohistochemical staining of KDM2A in iWAT and eWAT. (**B**,**C**) Immunofluorescence to detect KDM2A and FABP4 proteins by KDM2A antibody (red) and FABP4 antibody (green), respectively. Nuclei visualized with DAPI (blue) in mice inguinal white adipocytes (**B**) and epididymal white adipocytes (**C**). A merged image is shown in the panel on the far right. DM 13d: the cells were treated by differentiation medium for 13 days. Scale bar: 20 µm. NC represents negative control, IHC represents immunohistochemical staining, HE represents hematoxylin–eosin staining.

Furthermore, immunohistochemical staining and immunofluorescence staining were performed to reveal the localization of mouse KDM2A in adipose tissue (Figure 3A), inguinal white adipocytes (IWA) (Figure 3B) and epididymal white adipocytes (EWA) (Figure 3C). As shown in Figure 3A, immunohistochemical (IHC) staining results show that KDM2A was expressed in both iWAT and eWAT, and localized in the nucleus of adipocytes. Fatty-acid-binding protein 4 (FABP4), as a member of the lipid-binding protein superfamily, is believed to be a transporter protein that binds intracellular fatty acids with high affinity and transports them to the nucleus [18]. We found that FABP4 was expressed in the cytoplasm during the preadipocyte stage, and it gradually transferred from the cytoplasm to the nucleus with the adipocyte differentiation, expressed in both the nucleus and the cytoplasm to some extent (Figure 3B,C). This result is consistent with the findings of Yonekura et al. and Ayers et al. [19,20]. Interestingly, the KDM2A protein was always located in the nucleus, whether in preadipocytes or in mature adipocytes at the stage of terminal differentiation (Figure 3B,C).

### 2.4. Knockdown of KDM2A Inhibits the Proliferation of 3T3-L1 Preadipocytes

**Figure 4:**
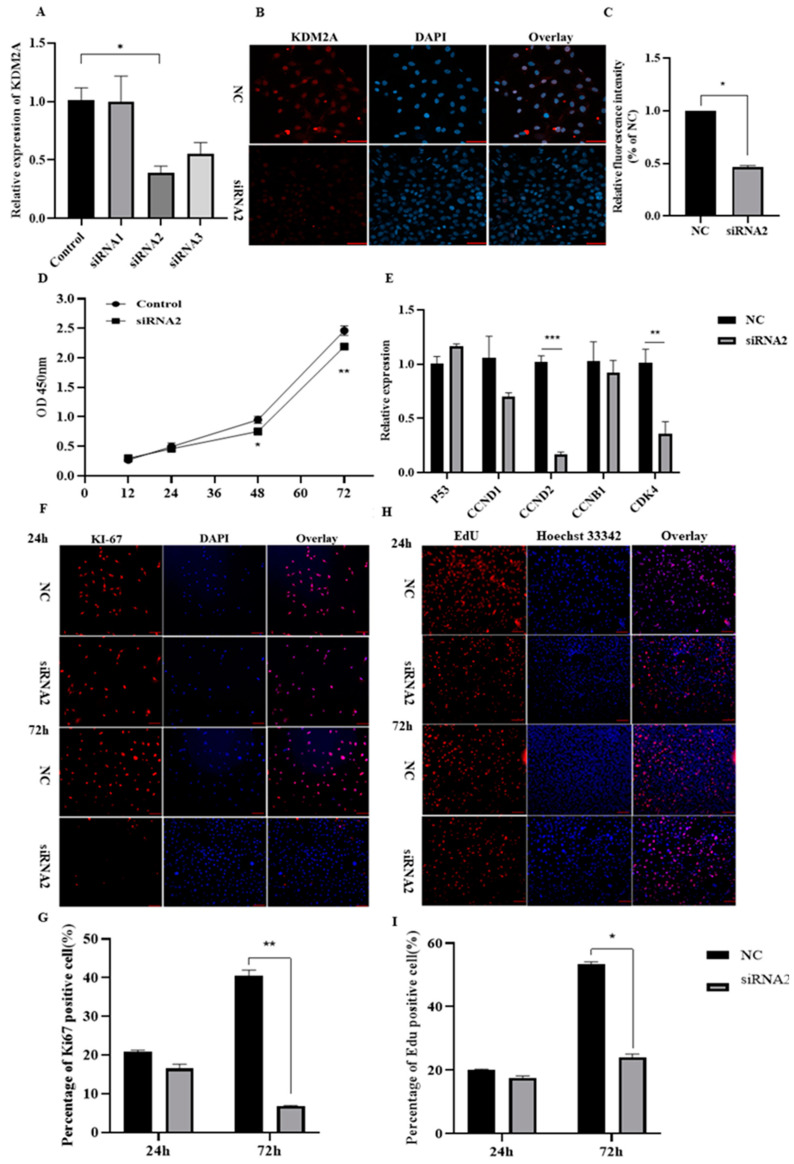
KDM2A loss of function inhibits the proliferation of 3T3-L1 preadipocytes. (**A**) The KDM2A mRNA expression level after transfection with siRNA1, siRNA2, siRNA3 and negative control (NC). (**B**,**C**) The images of immunofluorescence staining of KDM2A (**B**) and quantitative analysis of fluorescence intensity (**C**) at 24 h after transfection with siRNA2. (**D**) Cell proliferation was examined by CCK-8 analysis. (**E**) The mRNA levels of P53, CCND1, CCND2, CCNB1 and CDK4 were determined by qPCR. (**F**) The immunofluorescence staining of Ki67: red represents Ki67-positive cells and blue represents nucleus labeled by DAPI. (**G**) The percentage of Ki67-positive cells in siRNA2 treatment and NC groups. (**H**) The proliferation capacity of preadipocytes was examined by the EdU assay: red represents EdU staining and blue represents cell nucleus labeled by Hoechst 33342. (**I**) The percentage of EdU-positive cells in siRNA2 treatment and negative control group. Scale bar: 20 μm. N = 4; data are presented as mean ± SEM, * *p* < 0.05, ** *p* < 0.01, *** *p* < 0.001.

To assess the effect of KDM2A in cellular proliferation, 3T3-L1 preadipocytes were transfected with three individual siRNAs targeted for KDM2A, namely siRNA1, siRNA2 and siRNA3. As shown in Figure 4A, the siRNA2 and siRNA3 groups, respectively, downregulated the mRNA level of KDM2A by ~60% and ~50% to counterparts of the control, while siRNA1 did not have knockdown efficiency for the KDM2A gene. In accordance with the mRNA level, immunofluorescence staining and its quantitative analysis showed that the protein level of KDM2A declined by ~50% in the siRNA2 group to that of the control (Figure 4B,C), which indicated that the expression efficiency of siRNA2–KDM2A was sufficient for further analysis. Subsequently, the cell counting kit-8 (CCK-8) analysis showed that the knockdown of KDM2A significantly inhibited preadipocyte proliferation at 48 and 72 h after siRNA2 transfection, indicated by the reduced OD value at 450 nm in the siRNA2 group (Figure 4D). In compliance with the CCK-8 data, the KDM2A loss of function decreased the mRNA levels of proliferation-related genes, including cyclin D2 (CCND2) with ~80% and cyclin-dependent kinase 4 (CDK4) with ~60% lower than those of the control (Figure 4E). In addition, Ki67 immunofluorescence staining was carried out to confirm this phenomenon. The images and statistical analysis exhibited that siRNA2 treatment dramatically reduced Ki67-positive cells by ~80% at 72 h after transfection, compared to that of the control (Figure 4F,G). Furthermore, EdU staining analysis also showed that the proliferation capacity of 3T3-L1 preadipocytes was significantly inhibited in the siRNA2 group at 72 h, compared to that of the control (Figure 4H,I). Taken together, this evidence strongly demonstrates that the knockdown of KDM2A suppressed the proliferation of 3T3-L1 preadipocytes.

### 2.5. Knockdown of KDM2A Promotes 3T3-L1 Preadipocyte Differentiation

**Figure 5:**
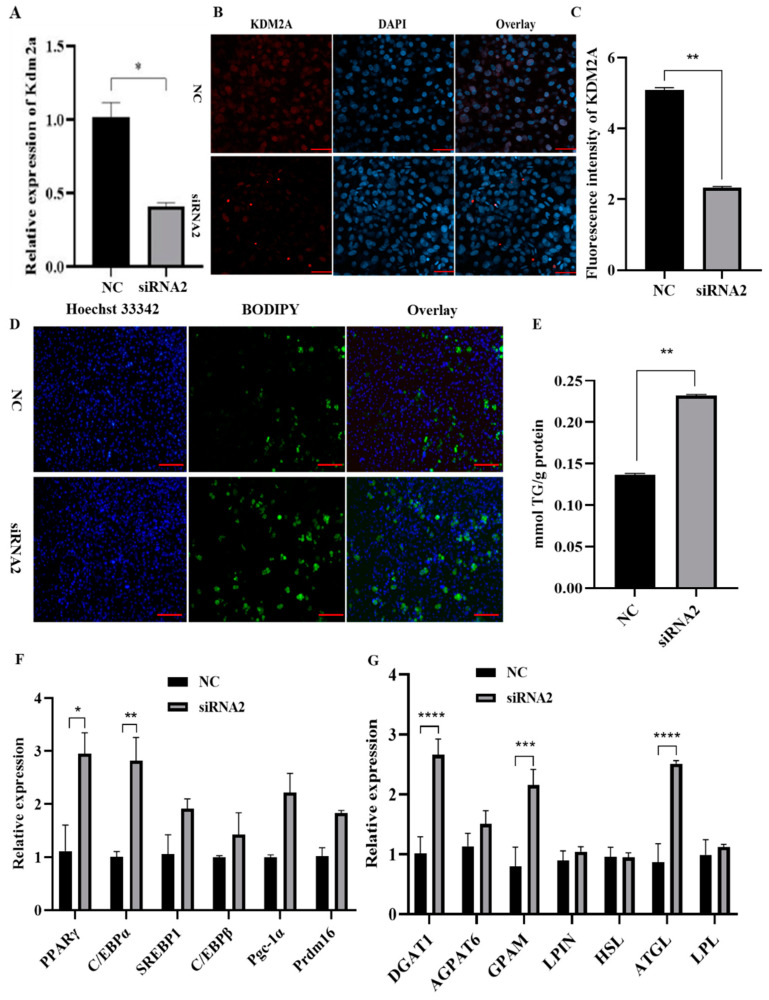
Knockdown of KDM2A promotes the differentiation of 3T3-L1 adipocytes. (**A**,**B**) The KDM2A mRNA expression level (**A**) and its immunofluorescence staining (**B**) after transfection and induction of differentiation at day 4: red represents KDM2A staining, and blue represents cell nuclei stained by DAPI. Scale bar: 20 μm. (**C**) Relative fluorescence intensity analysis of KDM2A. (**D**) BODIPY staining was performed after transfection and induction of differentiation at day 4: blue fluorescence labeling of the nucleus and green fluorescence labeling of the lipid droplets. Scale bar: 50 μm. (**E**) Determination of triglyceride (TG) content in 3T1-L1 adipocytes between KDM2A knockdown and NC groups. (**F**,**G**) The mRNA expression levels of transcriptional regulators (**F**) and triglyceride synthesis and lipolysis-related genes (**G**) in both KDM2A knockdown and NC groups. * *p* < 0.05, ** *p* < 0.01, *** *p* < 0.001, **** *p* < 0.0001.

We next examined whether the siRNA-mediated KDM2A knockdown affects the differentiation of 3T3-L1 preadipocytes (Figure 5). It was found that the mRNA level of KDM2A was significantly lower in the siRNA2-transfected group than in the NC group (Figure 5A). Consistently, immunofluorescence staining of KDM2A also showed that the protein level of KDM2A was significantly downregulated (Figure 5B,C). Subsequently, BODIPY staining showed that the knockdown of KDM2A increased lipid droplet accumulation significantly (Figure 5D). In compliance with morphology observations, the intracellular triglyceride (TG) content was measured using a TG assay kit, and the data show that the interference of KDM2A significantly elevated the TG content to ~1.7-fold of the control (Figure 5E). At the molecular level, we further analyzed the mRNA levels of adipogenic transcriptional regulators using qPCR, and the data show that the siRNA2 treatment significantly upregulated the levels of the peroxisome proliferator-activated receptor γ (PPARγ) and the CCAAT enhancer binding protein α (C/EBPα), while the mRNA levels of PPARγ coactivator 1α (Pgc-1α), CCAAT enhancer binding protein beta (C/EBPβ), sterol regulatory element-binding protein 1 (SREBP1) and PRD1-BF1-RIZ1 homologous domain-containing 16 (Prdm16) were not significant between the siRNA2 treatment and the NC group, with an increased trend (Figure 5F). Moreover, the TG synthesis-related genes, diacylglycerol O-acyltransferase-1 (DGAT1) and glycerol-3-phosphate acyltransferase, mitochondrial (GPAM), were largely elevated in the KDM2A knockdown groups (Figure 5G) compared with the control. However, the mRNA levels of 1-acylglycerol-3-phosphate O-acyltransferase 6 (AGPAT6) and lipid phosphate phosphohydrolase (LPIN) were not significant between the siRNA2 and NC groups (Figure 5G). Therefore, these data clearly demonstrate that the KDM2A loss of function promoted the differentiation of 3T3-L1 preadipocytes.

### 2.6. Deletion of KDM2A Promotes Primary Adipocytes Differentiation

**Figure 6:**
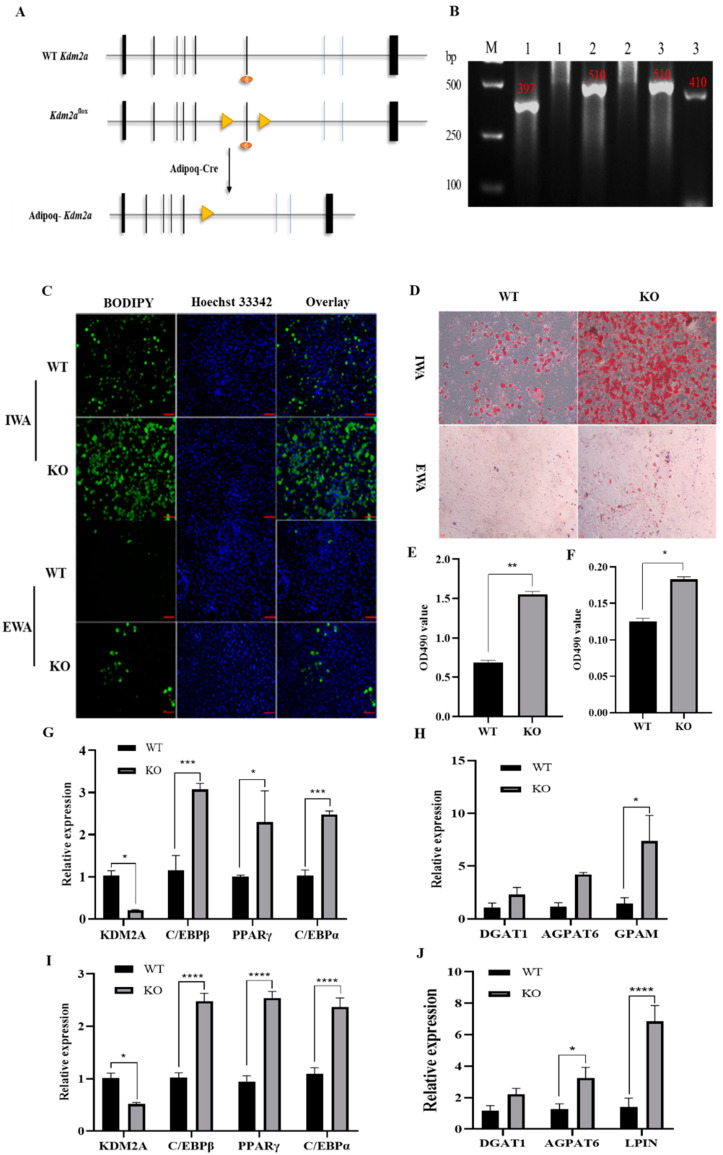
Knockout of KDM2A promotes the differentiation of primary adipocytes. (**A**) Schematic illustration of the Adipoq-Cre-mediated deletion of KDM2A mouse knockout strategy. Vertical lines represent exons and triangles represent the Loxp site. (**B**) Genotypic characterization of mice by gel electrophoresis. The wild-type (WT) band is 397 bp, the KDM2A^Flox/flox^ band is 510 bp and the Adipoq-Cre^+^ band is 410 bp. #1 is Adipoq-Cre^−^/KDM2A^−/−^ (WT), #2 is Adipoq-Cre^−^/KDM2A^flox/flox^ and #3 is Adipoq-Cre^+^/KDM2A^flox/flox^ (KO). (**C**) IWA and EWA were stained with BODIPY dye at day 10 after inducing differentiation: blue fluorescence labeling of nucleus and green fluorescence labeling of lipid droplets. (**D**) IWA and EWA were stained with Oil Red O at day 10 after adipogenic differentiation. (**E**,**F**) Quantitative analysis of Oil Red O staining in IWA (**E**) and EWA (**F**) from WT and KO mice. (**G**–**J**) The mRNA levels of differentiation transcriptional regulators and TG synthesis genes in IWA (**G**,**H**) and EWA (**I**,**J**). N = 4; data are presented as mean ± SEM, * *p* < 0.05, ** *p* < 0.01, *** *p* < 0.001, **** *p* < 0.0001, Scale bar: 50 µm.

To genetically elucidate the role of KDM2A in adipose tissues, we established a mouse model of adipose-tissue-specific knockout KDM2A mediated by Adipoq-Cre, which was widely used for knockout or insertion gene restriction in adipocytes [21,22]. In our case, in Adipoq-Cre^+^/KDM2A^flox/flox^ mice (abbreviated as Adipoq-KDM2A^f/f^, KO), the sixth exon of the KDM2A gene was knocked out (Figure 6A). Genotyping analysis showed that the genotypes of #1, #2 and #3 were Adipoq-Cre^-^/KDM2A^−/^^−^, Adipoq-Cre^-^/KDM2A^flox/flox^ and Adipoq-Cre^+^/KDM2A^flox/flox^, respectively (Figure 6B). Of these, #1 and #3 were named the wild-type (WT) and KO mouse, respectively (Figure 6B). We next isolated iWAT and eWAT stromal vascular fraction (SVF) cells from WT and KO mice, which were retained in the adipogenic induction program for 10 days. BODIPY staining results showed that the number of lipid-droplet-loaded positive cells in KDM2A KO white adipocytes from both iWAT and eWAT was significantly increased compared with those of the WT mouse (Figure 6C). Consistently, Oil Red O dye in inguinal white adipocytes (IWA) from the KO group was significantly higher than that in the WT group, and the lipid content was elevated with ~2-fold changes to the counterpart in the WT group (Figure 6D,E). Reliably, a similar result was also observed in epididymal white adipocytes (EWA) (Figure 6D,F). At the molecular level, the ablation of KDM2A significantly upregulated the mRNA levels of the adipogenic transcriptional factors, C/EBPβ with ~3.5-fold, PPARγ with ~2.5-fold and C/EBPa with ~3-fold changes in the inguinal KO white adipocytes compared to those of the WT (Figure 6G). In addition, the deletion of KDM2A increased the expression of TG synthesis-related genes (Figure 6H). In epididymal white adipocytes, the similar expression trends of the aforementioned measured genes were observed (Figure 6I,J). Summarily, these data indicate that the ablation of KDM2A facilitated the differentiation of primary adipocytes.

### 2.7. Knockdown of KDM2A Promotes Adipogenesis by Affecting Expression and Nuclear Translocation of PPARγ

**Figure 7:**
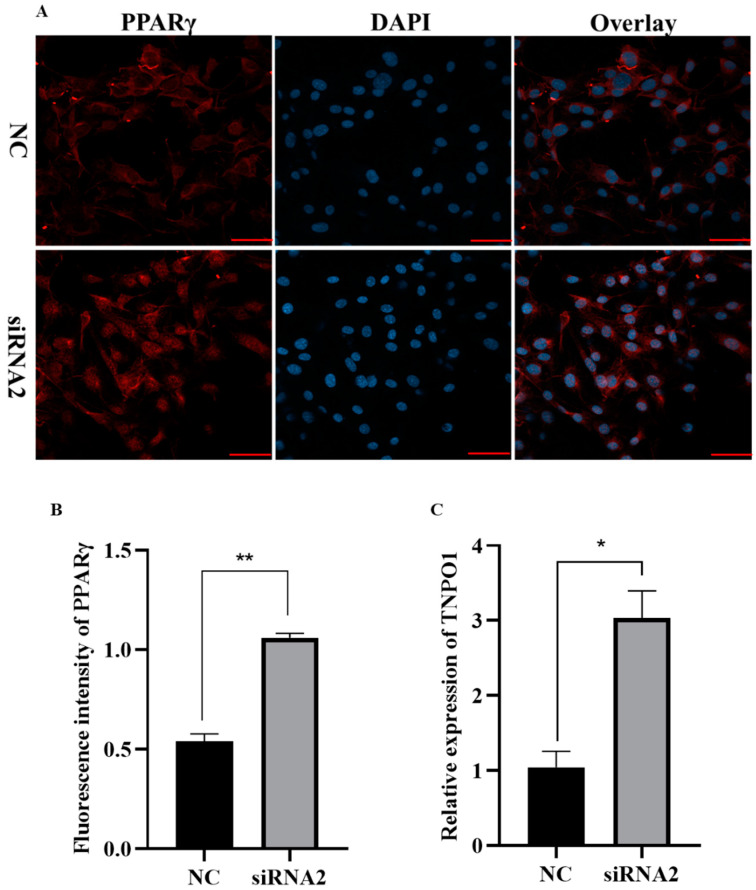
Knockdown of KDM2A promotes adipogenesis by affecting expression and nuclear location of PPARγ. (**A**) The immunofluorescence staining of PPARγ at day 4 after transfection and induction of differentiation. (**B**) Relative fluorescence intensity analysis of PPARγ. (**C**) The mRNA expression level of TNPO1. N = 4; data are presented as mean ± SEM, * *p* < 0.05, ** *p* < 0.01. Scale bar: 20 μm.

Previous ATAC-seq analysis showed that KDM2A^−/−^ in macrophages resulted in a significant gain of peaks at the PPARγ locus in three particular sites [23]. Our data show that the mRNA level (Figure 5F) and protein expression (Figure 7A,B) of PPARγ, as a crucial differentiation transcriptional regulator, were significantly upregulated after transfection and induction of differentiation for 4 d. Intriguingly, the knockdown of KDM2A impelled the translocation of PPARγ from the cytoplasm to the nucleus by immunofluorescence staining (Figure 7A). It was reported that the formation of the PPARγ2/Transportin 1 (TNPO1) complex through the disulfide bond in the cytoplasm leads to the nuclear translocation of PPARγ [24]. Therefore, we detected the mRNA level of TNPO1 and found it remarkably upregulated in the siRNA-KDM2A group (Figure 7C). Thus, these results suggest that the loss of function of KDM2A may occur through the upregulation of its transcription and nuclear translocation to promote adipogenesis.

## 3. Discussion

The post-translational modification of histones, especially histone methylation, regulates gene expression by affecting the chromatin structure, and this has become a fascinating topic in epigenetic research [25]. KDM2A is an important regulator of epigenetic modification. We report that KDM2A is a negative regulator of adipocyte differentiation; both the knockdown and knockout of KDM2A suppresses the proliferation and promotes the differentiation of adipocytes. Interestingly, the knockdown of KDM2A upregulates the transcription of PPARγ and promotes its nuclear translocation by TNPO1, and subsequently facilitates adipocyte differentiation. Our research extends the molecular mechanisms and metabolic networks of adipose tissue deposition to some extent.

The KDM2A loss of function inhibits the proliferation of 3T3-L1 preadipocytes, and this is assessed using three biased techniques (CCK-8 assay, EdU and Ki67 staining). It is well known that the occurrence of cell cycle regulation and apoptosis are the classical molecular mechanisms for cell proliferation and growth [26,27,28]. Consistent with our finding, the knockout of KDM2A in HEK293T cell lines significantly inhibited this cell proliferation through the downregulation of TGF-β signaling, leading to more cells being blocked in the G2/M phase [29]. In gastric cancer, the upregulation of KDM2A by LINC00460, as a molecular sponge for miR-342-3p targeting KDM2A, promoted the proliferation, migration and invasion of gastric cancer cells [30]. However, Liu et al. found that the knockdown of KDM2A using small hairpin RNA (shRNA) facilitated multiple myeloma (MM) cell proliferation [31]. These reports suggested that the exact roles and molecular mechanism in cell proliferation may be specific due to the difference in cell types.

The knockdown of KDM2A promoted the differentiation of adipocytes, which was characterized by increased adipocyte lipid accumulation. At the molecular level, adipocyte differentiation is a highly ordered process regulated by transcription factors, such as C/EBPα, C/EBPβ and PPARγ [32,33]. Recent studies have shown that in KDM2A knockout in macrophages, the level of H3K36me2 at the PPARγ locus is significantly enhanced, making macrophages inclined to M2 polarization, thus preventing mice from obesity and insulin resistance induced by a high-fat diet [23]. qPCR analysis also showed that KDM2A knockdown promoted the mRNA level of C/EBPα and PPARγ, as well as the genes related to TG synthesis, including DGAT1, GPAM and AGPAT6. Therefore, PPARγ is regarded as a candidate target by KDM2A.

Generally, PPARγ is translated in the cytoplasm; the translocation from the cytoplasm to the nucleus is a necessary step for role function. Transportin 1 (TNPO1), as an important karyopherin-β family member, transports many RNA-binding proteins that are involved in RNA processes or gene transcription into the nucleus [34]. Toshiaki et al. found that the formation of PPARγ could interact with TNPO1 through disulfide bonds to form a protein complex in the cytoplasm, leading to the nuclear translocation of PPARγ [24]. Interestingly, this was similar to our data. The KDM2A loss of function upregulated the expression of PPARγ and induced its nuclear translocation, accompanied by an elevation of the TNPO1 level. However, the crosstalk details of KDM2A/PPARγ/TNPO1 remain to be illustrated in further experiments.

In conclusion, our data indicate that KDM2A is a negative regulator of adipocyte differentiation. The upregulation of TNPO1 promotes adipocyte differentiation and lipid accumulation, which provides new insight into the molecular mechanism of lipid deposition in animals.

## 4. Materials and Methods

### 4.1. Animals and Cell Line

All the experiments were performed under the approval of the Animal Care and Use Committee of Southwest Minzu University. The mice were housed and maintained in the animal facility with free access to water and standard rodent chow food. It was light from 7:00 a.m. to 19:00 p.m. and dark from 19:00 p.m. to 07:00 a.m., and the temperature was controlled at approximately 25 °C. The *Adipoq*-Cre(stock # 010803)mice were donated by Professor Gongshe Yang from Northwest A&F University, and the 3T3-L1 cell line was donated by Dr. Zhang Bin from the Institute of Medicinal Plant Development, Chinese Academy of Medical Sciences and Peking Union Medical College. Additionally, the KDM2A^flox/flox^ mice were purchased from Saiye Biotechnology Co., Ltd. (Suzhou, China) PCR genotyping (Sigma-Aldrich, St. Louis, MO, USA) was performed as described by the supplier.

### 4.2. Primary Adipocyte Isolation, Culture and Differentiation

Primary WAT stromal vascular fraction (SVF) cells were isolated using collagenase-type I (Sigma, C0130, USA) digestion, followed by density separation [18]. Briefly, inguinal white adipose tissue (iWAT) and epididymal adipose tissue (eWAT) were minced and digested in 1 mg/mL collagenase at 37 °C for 60 min, then the digestions were terminated with DMEM (Gibco, C11995500BT, Grand Island, NY, USA) containing 20% FBS (AlphaCell, 100061, Shenzheng, China) and 1% P/S (Biosharp, BL505A, Hefei, China). Thereafter, the digestions were centrifuged for 10 min, 1700× *g* at room temperature. The sediment was re-suspended in DMEM supplemented with 20% FBS and diluted to a final concentration of 10^6^ cells/mL. These cells were cultured at 37 °C in a humidified atmosphere containing 5% CO_2_.

### 4.3. BODIPY and Oil Red O Staining

The cultured cells were washed three times with PBS (phosphate-buffered saline), and BODIPY (final concentration: 2 μM) and Hoechst 33342 (final concentration 10 µg/mL) dyes were added for 30 min. Then, the cells were washed three times with PBS, PBS was added and pictures were taken. For Oil Red O staining, the cells were fixed with 4% paraformaldehyde (PFA) for 15–20 min, and the Oil Red O working solutions were added containing 6 mL Oil Red O stock solution (5 g/L in isopropanol) and 4 mL ddH_2_O for 30–45 min. After staining, the pictures were captured by an Olympus CKX41. All images of the control group and the experimental group were taken at the same parameters.

### 4.4. Immunofluorescence Staining

For immunofluorescence staining, the cells were fixed with 4% PFA (Biosharp, BL539A, China) for 10–15 min, washed three times with PBS and then incubated with 100 mM glycine for 5–10 min to terminate the PFA. A block solution containing 5% goat serum, 2% BSA, 0.2% Triton X-100 and 0.1% sodium azide PBS solution was closed at room temperature for 30 min. Cells were incubated with a primary anti-KDM2A antibody (Abcam, EPR18602, UK), mouse anti-FABP4 antibody (Bioss, bsm-51247M, Shanghai, China), anti-Ki67 rabbit pAb (Wanlei, WL01384a, Shenyang, China) and anti-PPARγ rabbit pAb (Wanlei, WL01800, China) at 1:500 dilution in a blocked solution at 4 °C overnight. Alexa FluorTM 568 goat anti-rabbit igG (Invitrogen, A11011, Carlsbad, CA, USA) and goat anti-mouse IgG/FITC (Bioss, bs-0296G-FITC, Shanghai, China) conjugated secondary antibodies were used at a dilution of 1:500 and 1:1000 at room temperature for 1 h, respectively. All the pictures were taken under a Zeiss confocal microscope.

### 4.5. Total RNA Extraction and Quantitative Real-Time PCR (qPCR)

Total RNA was extracted from adipocytes or tissues using Trizol (TAKARA, 9109, Nojihigashi, Japan) reagent according to the manufacturer’s instructions. RNA was treated with RNase-free DNase I to remove genomic DNA. The purity and concentration of the total RNA were measured by Nanodrop ND-1000 (Thermo Fisher, Waltham, MA, USA). Ratios of absorption (260/280 nm) of all samples were between 1.9 and 2.1. Next, the total RNA (1000 ng) was reverse transcribed into cDNA using a PrimeScript^TM^ RT reagent kit with gDNA Eraser (Takara, RRO47A, Nojihigashi, Japan). qPCR was carried out with a Bio-Rad CFX96 PCR System using SYBR qPCR Master Mix (Vazyme, Q712-00, Chengdu, China) and gene-specific primers (Table S1). The 2^−∆∆CT^ method was used to analyze the relative changes in gene expression normalized against RPL13a as the reference gene [35].

### 4.6. Chemical Synthesis of siRNA

Three gene-specific siRNAs for KDM2A were designed online and synthesized according to the sequence of mouse KDM2A (NM_001001984.2); namely, siRNA1-KDM2A (5′-GCCAACGAAUUGAGCUCAATT-3′), siRNA2-KDM2A (5′-GCUCUGAUUGCUGAUGUAATT-3′) and siRNA3-KDM2A (5′-GGACAUCCAUUGUGCCCAATT-3′). A negative control was provided by GenePharma (5′-UUCUCCGAACGUGUCACGUTT-3′).

### 4.7. Cell Transfection

The 3T3-L1 adipocytes were seeded in 24-well plates or 35 mm culture dishes, and 90 pM siRNA–KDM2A and the negative control (NC) (Genepharma, Shanghai, China) were transfected into cells of 30–40% density using Lipofectamine^TM^ 3000 Reagent Protocol (Invitrogen, L3000-015, USA) and Opti-MEM (Gibco, 31985-070, USA) culture medium according to manufacturer’s protocol. After 24 h, the cells were collected to detect the proliferation. For adipogenic differentiation, the cells were transfected when the density of the 3T3-L1 adipocytes reached 50–60%. When the transfected cells grew to fusion, adipogenic differentiation was initiated by changing the differentiation medium.

### 4.8. EdU Staining

For this assay, 10 µM 5-ethynyl-2′-deoxyuridine (Beyotime, C0078S, Shanghai, China) was added into the growth medium and incubated for 2 h. Fixation, penetration and EdU staining procedures were performed in accordance with the manufacturer’s instructions. Hoechst 33342 (Beyotime, C1022, Shanghai, China) was used for nuclear staining at a concentration of 5 µg/mL for 10 min. Finally, the cells were captured by the ZEISS Laser confocal microscope and the positive rate was represented as EdU-positive cells/Hoechst 33342 cells.

### 4.9. CCK-8 Detection

The 3T3-L1 adipocytes were planked in 96-well plates at 5 × 10^3^ cells/well in 100 μL of growth medium. The CCK-8 kit (AbMole, Shanghai, China) was used to detect cell proliferation according to the manufacturer’s instructions at 12, 24, 48 and 72 h after treatment with siRNA2-KDM2A.

### 4.10. Statistical Analysis

Statistical analysis was performed using the GraphPad Prism 8.3 software. Multiple comparisons were performed using a one-way ANOVA and a two-way ANOVA. Differences were considered significant at *p* < 0.05 (* *p* < 0.05, ** *p* < 0.01). All experimental data are presented as mean ± SEM.

## Supplementary Materials

The following are available online at https://www.mdpi.com/article/10.3390/ijms22189759/s1.
