# Supplementary material for: Ablation of KDM2A Inhibits Preadipocyte Proliferation and Promotes Adipogenic Differentiation
Source: Int J Mol Sci. 2021 Sep 9;22(18):9759. doi: 10.3390/ijms22189759 (PMC8467897; doi:10.3390/ijms22189759)
Supplement: Supplementary file 1 [file ijms-22-09759-s001.zip › supplementary materials.pdf]

Table S1. Primers for quantitative real-time PCR (qPCR).

Genes	Sequence	Size	Number
Kdm2a-F	GCCAAGGCACTTGAAAGAAA	104bp	XM_006531722.4
Kdm2a-R	AGCAGCCTCGAACACTCATT		
P53--F	CAACAAATGCTGGCTACTAAGGA	198bp	NM_173378.2
P53-R	CACGAGTTTTCCGTTGCTCA		
CCND1-F	TAGGCCCTCAGCCTCACTC	80bp	NM_001379248.1
CCND1-R	CCACCCCTGGGATAAAGCAC		
CCND2-F	GAGTGGGAACTGGTAGTGTTG	154bp	XM_036165787.1
CCND2-R	CGCACAGAGCGATGAAGGT		
CCNB1-F	CTTGCAGTGAGTGACGTAGAC	94bp	NM_172301.3
CCNB1-R	CCAGTTGTCGGAGATAAGCATAG		
CDK4-F	CTGAACCGCTTTGGCAAGAC	243bp	XM_006509968.2
CDK4-R	GCCCTCTCTTATCGCCAGAT		
Pgc1α-F	GGATTGAAGTGGTGTAGCGAC	58bp	XM_036164894.1
Pgc1α-R	GCTCATTGTTGTACTGGTTGGA		
C/EBPβ-F	CGCCTTATAAACCTCCCGCT	167bp	XM_021194199.2
C/EBPβ-R	TGGCCACTTCCATGGGTCTA		
PPARγ-F	AAGAAGCGGTGAACCACTGA	154bp	XM_036165927.1
PPARγ-R	GGAATGCGAGTGGTCTTCCA		
SREBP1-F	GTTTCCGGGGAACTTTTCCT	165bp	NM_001313979.1
SREBP1-R	GAGCTGGAGCATGTCTTCGAT		
C/EBPα-F	GAGGGGAGGGACTTAGGTGT	171bp	NM_001287514.1
C/EBPα-R	TGCCCCCATTCTCCATGAAC		
prdm16-F	CAGCACGGTGAAGCCATTC	87bp	XM_036164423.1
prdm16-R	GCGTGCATCCGCTTGTG		
GPAM-F	CAAATAGGCCTCTGGAGGAGC	194bp	NM_001356285.1
GPAM-R	GATCTTTTGCCAGGCTCCTGAT		
DGAT1-F	ACGGATCATTGAGCGTCTCT	161bp	NM_010046.3
DGAT1-R	TGACAGACTCAGCATTCCACC		

LPIN-F	GCTCGTGAATCCTCTTGGTTCAG	198bp	XM_011243815.4
LPIN-R	GGACACCCATCTTGCCGAAG		
AGPAT6-F	AGCTTGATTGTCAACCTCCTG	212bp	XM_011242104.4
AGPAT6-R	CCGTTGGTGTAGGGCTTGT		
HSL-F	ACTCAGACCAGAAGGCACTA	113bp	XM_030242180.1
HSL-R	TAGTTCCAGGAAGGAGTTGA		
ATGL-F	CCTTAGGAGGAATGCCCTGC	124bp	NM_025802.3
ATGL-R	CTCCAGCGGCAGAGTATAGG		
LPL-F	GCTGGGCCTAACTTTGAGTATG	64bp	XM_021169248.2
LPL-R	CAAAATCAGCGTCATCAGGAGAA		
RPL13a-F	GAGGTCGGGTGGAAGTACCA	71bp	XM_021166430.2
RPL13a-R	TGCATCTTGGCCTTTTCCTT		
